# The Characterization of the Repertoire of Wheat Antigens and Peptides Involved in the Humoral Immune Responses in Patients with Gluten Sensitivity and Crohn's Disease

**DOI:** 10.5402/2011/950104

**Published:** 2011-10-27

**Authors:** Aristo Vojdani

**Affiliations:** Immunosciences Laboratory, Inc., 822 S. Robertson Boulevard, Suite 312, Los Angeles, CA 90035, USA

## Abstract

Intestinal T cells from gluten sensitivity/celiac disease patients respond to a heterogeneous array of peptides. Our study extended this heterogeneity to humoral immune response to various wheat proteins and peptides in patients with gluten sensitivity or Crohn's disease. IgG and IgA antibodies in sera from those patients and healthy control subjects were measured against an array of wheat antigens and peptides. In gluten-sensitive patients, IgG reacted most against transglutaminase, prodynorphin, wheat extract, and *α*-, *γ*-, and *ω*-gliadin; IgA reacted most against wheat then transglutaminase, glutenin, and other peptides. In the sera of Crohn's disease patients, IgG reacted most against wheat and wheat germ agglutinin then transglutaminase, prodynorphin, *α*-, and *γ*-gliadin; IgA reacted foremost against prodynorphin then transglutaminase and *α*-gliadin. These results showed a substantial heterogeneity in the magnitude of IgG and IgA response against various wheat antigens and peptides. Measurements of IgG and IgA antibodies against such an array of wheat peptides and antigens can enhance the sensitivity and specificity of serological assays for gluten sensitivity and celiac disease and may also detect silent celiac disease or its overlap with inflammatory bowel disease.

## 1. Introduction

Despite the efforts of several laboratories to define relevant gluten epitopes, the characterization of the complete repertoire of peptides involved in the pathogenesis of celiac disease and associated disorders remains a daunting task because of the great heterogeneity of gluten proteins [1–4]. So far, several T cell stimulatory peptides from *α*-gliadin, *γ*-gliadin, and glutenins have been identified [1–4]. 

In a very recent study [5], intestinal T cells were isolated from 14 adults with celiac disease (CD) for recognition of 21 peptides derived from *α*-, *γ*-, *ω*-gliadins and glutenin. Results demonstrated that patients respond to a wide heterogeneous array of peptides; some recognized many peptides from single or multiple gliadin families, while others reacted to only one peptide. These results confirmed that a large number of gluten epitopes may be implicated in the development of gluten sensitivity, CD, and associated diseases. Indeed, a T cell line from a patient failed to recognize any of the 21 tested peptides. This suggests that other gliadin peptides and proteins are involved in the pathogenesis of gluten-sensitive enteropathy or CD [5]. T-cell responses of adult CD patients toward the overall 10 *α*-gliadin-derived peptides assayed indicated that they mainly focused on the 33-mer and its shorter forms, with the 17, 18, and 25-mer being the most frequently recognized by the T cell. 

In contrast, responses elicited by *γ*-gliadin-derived peptides were less focused than those induced by *α*-gliadin-derived peptides, most likely reflecting their more diverse sequences. Furthermore, the great majority of patients reacted to at least one *γ*-gliadin peptide and an overall half-recognized DQ2-*γ*-I. This frequent recognition of *γ*-gliadin peptides by intestinal T cells from individuals with CD suggests that their contribution to CD pathogenesis may be greater than what we had thought. They also found that intestinal T cell lines were frequently and strongly stimulated by the *ω*-gliadin-derived peptide, DQ2*ω*-I [4–6]. 

Understanding the hierarchy and consistency of epitopes is important, as recent studies have shown that immunodominant epitopes not only can aid in a better diagnosis, but also can have therapeutic applications for the induction of tolerance in several T cell-mediated diseases [7–9]. 

Conflicting data have been reported regarding the immunodominance of gluten peptides. For example, 50% of T cell lines derived from Dutch children and adults were reactive to peptides 33-mer and 13-mer [10]. This was consistent with the findings of Camarca et al., who also showed that 50% of T cell lines derived from CD patients recognized 33-mer of *α*-gliadin [5]. In contrast, 33-mer was universally recognized by HLA-DQ2^+^ in Norwegian CD patients [3, 11]. Overall, however, Camarca et al.'s study showed that there is a substantial heterogeneity in the intestinal T cell responses to *α*-, *γ*-, *ω*-gliadin and glutenin peptides and these peptides are the most active peptides that play a significant role in the pathogenesis of CD. 

Having observed the heterogeneity of intestinal T-cell responses to gluten peptides, we wanted to see whether or not this immune reaction could similarly be extended to humoral immune responses, in particular IgG and IgA antibody production against the repertoire of antigens and peptides associated with gliadin in patients with gluten sensitivity as well as in patients with Crohn's disease.

Crohn's disease and ulcerative colitis fall under the classification of inflammatory bowel disease (IBD). They are triggered by environmental factors including food and microbial antigens [12]. The serologic response in Crohn's disease includes antibodies against specific components of *Saccharomyces cerevisiae*, mycobacteria, bacteroides, and *Escherichia coli* [12–16]. In fact, the measurement of antibodies to baker's and brewer's yeasts directed against cell wall oligomannoside epitope (ASCA) have been proposed as a serological marker for Crohn's disease [17]. These antibodies have a sensitivity of 60%–70% for differentiating Crohn's disease from controls and a specificity of 80%–95% [12–18]. Due to overlapping symptomatology between celiac and Crohn's disease, ASCA antibodies were also measured in a group of patients with CD. High incidences of ASCA were reported in patients with gluten sensitivity enteropathy (GSE). The IgG and IgA antibodies in the sera of GSE patients provided proof of a systemic response against *Saccharomyces cerevisiae* that suggested a breakdown in oral tolerance against the yeast antigens [19, 20]. The high prevalence of ASCA in patients with celiac disease encouraged us to expand the aim of this study from humoral immune response against a repertoire of wheat antigens and peptides in celiac disease to patients with Crohn's disease. 

## 2. Materials and Methods

A whole-wheat antigen was prepared by combining water-soluble and alcohol-soluble proteins. Different gliadin peptides including *α*-gliadin-33-mer, -17-mer, *γ*-gliadin-15-mer, *ω*-gliadin-17-mer, glutenin-21-mer, gluteomorphin-16-mer, prodynorphin, transglutaminase (TG), and gliadin bound to TG, HPLC grade were synthesized by Bio-Synthesis Inc., (Lewisville, Tex, USA). Wheat germ agglutinin (WGA) was purchased from Sigma/Aldrich (Saint Louis, Mo, USA).

Forty-eight sera from healthy control subjects aged 18–65 were obtained from Innovative Research (Novi, Mich, USA). Commercially available sera of 24 patients with gluten sensitivity/celiac disease and 24 patients with Crohn's disease were purchased from The Binding Site (San Diego, Calif, USA), Inova (San Diego, Calif, USA), Trina International Nanikon (Switzerland), Diamedix (Fl, USA), and Innovative Research (Novi, Mich, USA).

### 2.1. Measurement of IgG and IgA by ELISA

Antigen and peptides were dissolved in methanol at a concentration of 1.0 mg/mL then diluted 1 : 100 in 0.1 M carbonate-bicarbonate buffer, pH 9.5, and 100 *μ*L each of wheat, *α*-gliadin-33-mer, *α*-gliadin-17-mer, *γ*-gliadin-15-mer, *ω*-gliadin-17-mer, glutenin-21-mer,  gluteomorphin-16-mer and prodynorphin, gliadin-bound transglutaminase, transglutaminase (TG), and WGA were added to rows 1–11 of a microtiter plate. Row no. 12 was coated with 100 *μ*L of 10 *μ*g/mL of human serum albumin and used as control. Plates were incubated overnight at 4°C and then washed three times with 200 *μ*L Tris-buffered saline (TBS) containing 0.05% Tween 20 (pH 7.4). The non-specific binding of immunoglobulins was prevented by adding 200 *μ*L of 2% bovine serum albumin (BSA) in TBS, and incubated overnight at 4°C. Plates were washed as mentioned previously, and then serum samples diluted 1 : 100 in 1% BSA in TBS containing 0.05% Tween 20 were added to duplicate wells and incubated for 1 hour at room temperature. 

Plates were washed, and then alkaline phosphatase goat antihuman IgG or IgA F(ab′)_2_ fragments (KPI, Gaithersburg, MD) optimal dilution of 1 : 400 for IgA and 1 : 800 for IgG in 1% BSA-TBS were added to each appropriate well; plates were incubated for an additional hour at room temperature. After washing five times with TBS-Tween buffer, the enzyme reaction was started by adding 100 *μ*L of 1 mg/mL paranitrophenylphosphate in diethanolamine buffer containing 1 mM MgCl_2_ and sodium azide (pH 9.8). The reaction was stopped 45 mins later with 50 *μ*L of 2 N NaOH. The optical density (OD) was read at 405 nm by the means of a microtiter reader. To exclude nonspecific binding, the ODs of the control wells coated with HSA (Row no. 12) were subtracted from all other wells. Sera from patients with celiac disease with known high titers of IgG and IgA against gliadin and transglutaminase peptides were used as positive controls.

### 2.2. Ethics

All samples were obtained from regulated and certified commercial providers who strictly maintain the anonymity of their sample donors and who are compliant with all required appropriate ethical practices.

### 2.3. Statistics

Statistics on Software (S.O.S.) version 2 was used for statistical analysis. Normal distribution was tested by the Kolmogorov-Smirnov one-sample test. One-way analysis of variance was performed by means of ANOVA. For post hoc analysis, the large sample *Z*-test was employed. Analysis of population variances was performed using the *F*-test. *P* values were used to determine levels of significance.

## 3. Results

### 3.1. Number of Patients and Tests

The data for IgG and IgA antibodies against an array of wheat antigens and peptides plus TG were derived from the sera of 48 healthy control subjects ages 18–65, 50% male and 50% female, with no history of GI disorder including gluten sensitivity and inflammatory bowel disease. For comparison, these antibodies were also measured in 48 sera which, based on elevations in gliadin and transglutaminase IgG, IgA (24 sera) and anti-Saccharomyces IgA (24 sera) were classified with the possibility of gluten sensitivity/celiac disease and Crohn's disease, respectively. The degree of positivity of these sera were confirmed using INOVA kits for gliadin, transglutaminase IgG, IgA and *Saccharomyces cerevisiae* (ASCA) IgA. Of the total number of serological tests, the 24 sera from patients with gluten sensitivity/celiac disease showed different degrees of antibody level with at least one out of four (gliadin IgG, IgA, transglutaminase IgG, IgA) tests being positive. The other 24 patients with Crohn's disease were ASCA-positive to varying degrees.

### 3.2. Prevalence of IgG and IgA Antibodies against Wheat and Various Gliadin Peptides in Sera of Healthy Control Subjects

We selected a large panel of peptides to represent *α*-, *γ*-, *ω*-gliadin, glutenin, gluteomorphin, dynorphin, TG, and gliadin bound to TG. In addition, since WGA has a capacity to bind to different cells, inducing production of anti-WGA antibody [21, 22], we included WGA in our antibody testing. 

In healthy control subjects, we found moderate elevation (ELISA OD 0.4–1.0) of IgG antibody against glutenin-21-mer in 11/48, gluteomorphin 10/48, wheat in 9/48 specimens, and for *α*-gliadin-33-mer, *ω*-gliadin-17, gliadin-TG, and WGA 1/48. IgG was not detected against *α*-gliadin-17, *γ*-gliadin-15, prodynorphin, and TG in any of the 48 control sera (see Table 1). The mean OD of IgG antibody against wheat and other associated antigens in healthy controls varied from 0.07 ± 0.08 for *γ*-gliadin 15-mer to 0.29 ± 0.18 for glutenin 21-mer (see Table 2). 

The IgA antibody was also measured against this array of peptides and antigens in healthy controls. Moderate elevation in IgA antibody was detected against *α*-gliadin-17-mer and glutenin-21-mer in 9 out of 48 sera and against wheat and gluteomorphin 5/48, prodynorphin 4/48, *α*-gliadin-33-mer, and *ω*-gliadin 2/48. The IgA antibody was not detected against *γ*-gliadin-15-mer, gliadin-TG, TG, and against WGA (see Table 1). The mean OD of IgA antibody against this array of antigens and peptides in healthy controls was as low as 0.06 ± 0.06 for gliadin + TG and as high as 0.25 ± 0.26 for *α*-gliadin-17-mer (Table 3). 

At the cutoff point of 0.39 OD or 3 SD above the ELISA background of wells coated with HSA in control sera, IgG antibody was detected in 23% against glutenin-21, 21% against gluteomorphin, and 19% against wheat. Against the other peptides or antigens, the IgG antibody was detected in only 2% of the tested specimens or not at all (Table 1).

The pattern of IgA antibodies against these antigens and peptides was different from IgG. The IgA antibody against *α*-gliadin-17 and against glutenin-21 was detected in 19%, followed by wheat and gluteomorphin (10%), prodynorphin (8%), (6%) and 4% against both *α*-gliadin-17-mer, and *ω*-gliadin-17. None of the sera from healthy controls showed elevation in IgA antibody against *γ*-gliadin-15, gliadin-TG, TG, and WGA (Table 1). 

### 3.3. Detection of IgG and IgA Antibodies against Wheat and Various Gliadin Peptides in the Sera of Patients with Gluten Sensitivity/Celiac Disease

The IgG antibodies against these antigens were measured in clinical specimens from patients with gluten sensitivity/celiac disease who were positive for gliadin, TG, or their combination.

**Figure 1:**
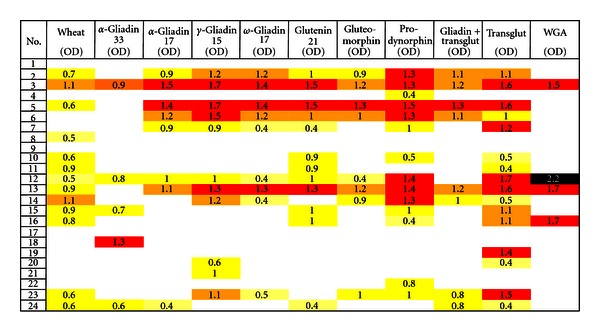
Prevalence of IgG expressed as optical density (OD) against wheat alone or in combination with gliadin, glutenin peptides, exorphins, gliadin-transglutaminase, transglutaminase, and WGA in sera of patients with gluten sensitivity/celiac disease.

We found four different profiles of peptides and antigen recognition by the sera of patients with CD. Results of these peptides and antigen recognition are illustrated in Figure 1 and Table 1. At ELISA, OD of 0.39 or 3 SD above the blank value IgG antibody was most reactive against TG in 16/24 specimens, then prodynorphin in 14/24, wheat in 13/24, glutenin in 12/24, *γ*-gliadin-15 in 11/24, *ω*-gliadin-17 in 10/24, gluteomorphin, *α*-gliadin-17, and gliadin-TG in 8/24, and against *α*-gliadin-33-mer 5 out of 24 specimens. Against WGA 4 out of 24 specimens were positive for IgG antibody.

Twelve out of 24 specimens (50%) in various intensities that showed a significant elevation of IgG antibody against wheat also exhibited elevation in the levels of this antibody against *α*, *γ*, *ω*-gliadins, glutenin, gluteomorphin, gliadin-TG, and WGA or their combinations (Figure 1).

Interestingly, the 12 specimens that reacted to wheat antigens and 3 or more different gliadin and glutenin peptides all produced strong response against tissue TG, while one specimen that reacted to wheat did not react with any other antigen. Of the remaining 11 specimens (46%) that did not react to wheat antigens, 4 did not react to other antigens, and the other 7 samples with various intensities were reactive against 1 to 8 different antigens or peptides (Figure 1). The mean OD of IgG antibody against 11 wheat and associated antigens varied from 0.18 ± 0.37 to 0.85 ± 0.76. Statistically, the differences between the mean ODs of IgG antibody against 9 out of 11 wheat-associated antigens in patients with gluten sensitivity/celiac disease versus healthy controls were significant (*P* < 0.0001 for TG to *P* < 0.0167 for *ω*-gliadin-17), with *P* < 0.1565 for gluteomorphin the least significant (Table 2).

**Figure 2:**
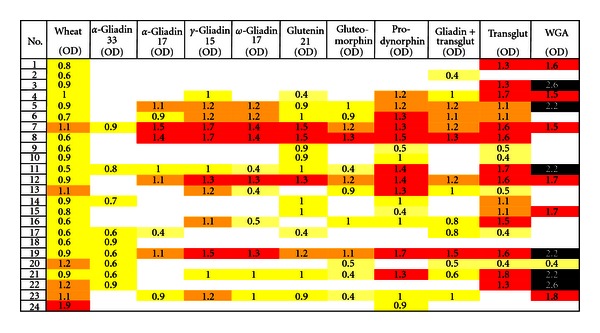
Prevalence of IgA expressed as optical density (OD) against wheat alone or in combination with gliadin, glutenin peptides, exorphins, gliadin-transglutaminase, transglutaminase, and WGA in sera of patients with gluten sensitivity/celiac disease.

The pattern of IgA antibodies against these same antigens and peptides was different from the pattern for IgG. All 24 specimens showed reactivity to more than one antigen or peptide. The most prominent reactions were against wheat and TG. Data summarized in Table 1 and Figure 2 shows that 24/24 (100%) and 20/24 (83%) samples reacted with IgA antibodies against wheat and TG, respectively, followed by prodynorphin with 17/24 (71%), glutenin-21 with 15/24 (63%), gliadin-TG 14/24 (58%), WGA 13/24 (54%), both gluteomorphin and *γ*-gliadin-15 with 12/24 (50%), *ω*-gliadin-17 11/24 (46%), and then both *α*-gliadin-17 and *α*-gliadin-33 with 9/24 (38%). Statistically, the differences between the mean ODs of IgA antibody against all of the wheat-associated antigens in patients with celiac disease versus healthy controls were significant, with 6 having *P* values of *P* < 0.0001, with the least significant being *P* < 0.0411 for *α*-gliadin-17 (Table 3).

### 3.4. Detection of IgG and IgA Antibodies against Wheat and Various Gliadin Peptides in the Sera of Patients with Crohn's Disease

**Figure 3:**
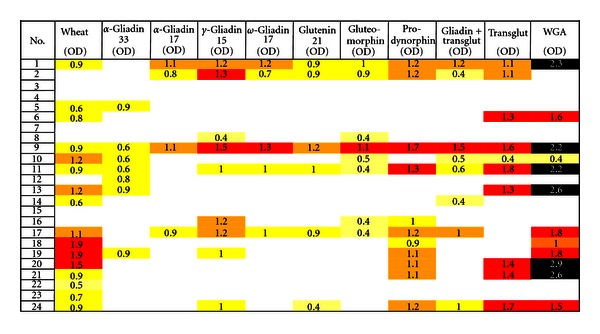
Prevalence of IgG expressed as optical density (OD) against wheat alone or in combination with gliadin, glutenin peptides, exorphins, gliadin-transglutaminase, transglutaminase, and WGA in sera of patients with Crohn's disease.

IgG and IgA antibodies against different wheat antigens and peptides, TG, gliadin bound to TG, and WGA were also measured in sera with IgA ASCA positive. For IgG antibody, at the 0.39 OD cutoff, 16 out of 24 (67%) of ASCA-positive specimens reacted with wheat, 12 specimens out of 24 reacted very strongly with WGA (50%), 11 with prodynorphin (46%), 10 with TG (42%), 9 with *γ*-gliadin-15 (38%), 8 with gluteomorphin and gliadin-TG (33%), 7 with *α*-gliadin-33 (29%), 6 with glutenin-21 (25%), 5 with *ω*-gliadin-17 (21%), and 4 with *α*-gliadin-17 (17%). Interestingly, all 12 WGA-reactive specimens also reacted with wheat antigens with or without the combination of gliadin peptides (Figure 3).

The mean ODs for IgG antibodies against various wheat and associated peptides and antigens in healthy controls were compared to those in patients with Crohn's disease, obtaining the most significant *P* values with *P* < 0.0002 for prodynorphin, TG and WGA, as well as the least significant *P* values *P* < 0.4744 for glutenin, as are shown in Table 1. 

**Figure 4:**
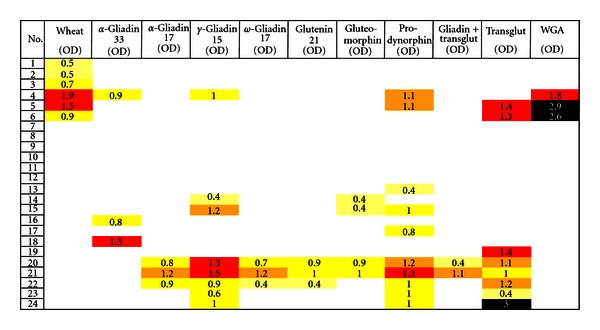
Prevalence of IgA expressed as optical density (OD) against wheat alone or in combination with gliadin, glutenin peptides, exorphins, gliadin-transglutaminase, transglutaminase, and WGA in sera of patients with Crohn's disease.

In comparison to IgG, the prevalence of IgA-positive specimens in IgA ASCA-positive samples was much lower. Overall, 10 out of 24 specimens (42%) reacted with prodynorphin, 8/24 (33%) against TG and *γ*-gliadin-15, 6/24 (25%) against wheat, followed by gluteomorphin with 4/24 (17%), *α*-gliadin-33, *α*-gliadin-17, *ω*-gliadin-17, glutenin-21, and WGA with 3/24 (13%), and 2/24 for gliadin-TG (8%). Six (25%) of the ASCA-positive samples did not exhibit any IgA antibody against the 11 tested wheat or associated antigens and peptides (Figure 4).

 The mean OD of IgA antibody against 11 tested wheat-associated antigens and peptides in the sera of patients with Crohn's disease are also shown in Table 3. Differences between the mean ODs of IgA antibody against 3 out of 11 tested antigens in healthy controls versus patients with Crohn's disease were significant (*P* < 0.0035 for prodynorphin, *P* < 0.0044 for *γ*-gliadin-15, *P* < 0.0047 for TG (Table 2)). 

The overall number and percentage of healthy controls versus patients' sera with elevated IgG and IgA antibody against wheat antigens and associated peptides are shown in Table 1. As shown in this table, the difference in percentage of individuals with elevated antibodies in healthy controls versus patients is very significant (*P* < 0.0004 for IgG antibody in the gluten-sensitive/celiac group, *P* < 0.0017 for IgG in the Crohn's group, and *P* < 0.0001 for IgA antibody in the gluten-sensitive group). While there is a significant overlap between IgG and IgA antibodies in both patients' groups, the percentage of IgA-reactive specimens against various tested antigens was the most significant in the gluten-sensitive group, followed by IgG presence in both the gluten-sensitive and Crohn's disease groups, with IgA reactivity against these antigens being the least significant in patients with Crohn's disease (Table 1). 

## 4. Discussion

A number of gluten peptides with a capacity to stimulate intestinal T-helper cells have been identified in CD patients by many researchers (2–6, 10, and 23–26). In a very recent study T cells isolated from CD patients were screened for recognition of 21 different peptides from *α*-, *γ*-, *ω*-gliadins and glutenins [5]. It was demonstrated that intestinal T cells from CD patients responded to a wide and heterogeneous array of peptides [5]. In some patients, many peptides from the *α*-gliadin family were recognized, while in others, only one peptide caused lymphocyte stimulation and interferon-*γ* production.  Furthermore, T-cell lines from one particular patient did not recognize any of the 21 tested peptides at all, while, overall, 86% of CD patients recognized a different array of peptides. It was concluded that other gliadin peptides not tested in the study could be relevant in some CD patients [5].

Although all these findings showed great heterogeneity in immunogenicity of gluten peptides for lymphocyte proliferation and IFN-*γ* production [2–6, 23–26], no attempt was made to measure heterogeneity in antibody response to various gluten and overall wheat proteins and peptides. In the present study, we screened the sera of patients with gluten-sensitivity/celiac disease and Crohn's disease for the presence of IgG and IgA antibodies against both alcohol- and water-soluble components of wheat, *α*-gliadin-33-mer, -17-mer, *γ*-gliadin-15-mer, *ω*-gliadin-17-mer, and glutenin-21-mer. 

The second category of peptides consisted of the opioids. Such peptides are called exorphins because of their exogenous origin and morphine-like characteristics. In some individuals, dietary exorphins are resistant to intestinal and enterobacterial proteinases; thus, gluteomorphins and dynorphins may be absorbed from the gut lumen into the bloodstream. Consequently, an immune response against the opioid peptides can result in peptide antibody production and regulation of opioid receptor binding capability [27, 28].

Thirdly, lectins were incorporated into this antibody array. WGAs are lectins, or carbohydrate-binding proteins, with a capacity to bind to many cells and tissue antigens, including intestinal brush borders. Lectins, bound to intestinal cells and other cell membranes, are known to induce toxic damage, inflammation, and autoimmunity [21, 22, 29].

Finally, earlier studies showed that gluten-sensitive patients develop IgG and IgA antibodies to gliadin and to the autoantigen called transglutaminase [30, 31]. These articles demonstrated that gliadin is the preferred substrate of transglutaminase and suggested that the interaction of gliadin and TG may result in the creation of new antigenic complexes [31, 32]. Indeed, in a different study [33], it was shown that at high molar excess gliadin peptides bind to six lysine residues of TG, forming isopeptide bonds. However, despite this demonstration of the molecular characterization of covalent complexes between tissue TG and gliadin peptides and discussion about its relevance in celiac disease, no attempt was made to measure antibodies against different gliadin peptides and their complex formation with TG. Therefore, we extended the measurement of IgG and IgA antibodies against this gliadin and TG complex as well. 

Similar to intestinal T-cell response, we demonstrated that humoral immune response to various wheat antigens and associated peptides are largely heterogeneous [5, 23]. Consistent with previous studies conducted with intestinal (T-cell) response against a heterogeneous array of wheat glutenin and *α*-, *γ*-, *ω*-gliadins, our results with IgG- and IgA-specific antibodies demonstrate that both sera with gluten-sensitivity/celiac disease and Crohn's disease and, to a much lesser degree, sera from healthy controls respond to a heterogeneous array of peptides and antigens.

In some cases, IgG and IgA antibodies were detected against wheat antigens alone or in combination with *α*-, *γ*-,  and  *ω*-gliadins and glutenin peptides, while in others, IgG or IgA were detected against one or more peptides without reacting to wheat antigens. This lack of humoral immune response to water- and alcohol-soluble components of wheat indicates that digestion of wheat proteins into various peptides and their deamidation by TG plays a significant role in their antigenicity. The selective deamidation of gliadin peptides and their complex formation with TG make them more specific B cell epitopes, which result in first IgA and then IgG production [31–33]. Indeed, IgA was detected in the great majority of patients with CD against wheat antigens (100%), followed by immune reaction against prodynorphin (71%), glutenin-21 (62%), gliadin-TG (58%), WGA (52%), and against other proteins and peptides between 37% and 50%, as seen in Table 1. 

In comparison with IgA, IgG was detected in the sera of celiac disease patients most prominently against TG, followed by prodynorphin, wheat extract, and then glutenin-21 mer (Table 1). The current methodology for diagnosing celiac disease is based on measuring IgG and IgA antibodies against gliadin and TG [30–38]. The specificity and sensitivity of these assays in patients with CD who exhibit abnormal histology (villous atrophy or flat mucosa) varies between 85% and 100% [34–38]. However, this specificity and sensitivity have not been established for patients with gluten sensitivity and patients with silent or atypical celiac disease who may have GI symptoms but normal villi. Autoantibodies can be detected in various diseases for a long period during which no clinical symptoms are present [39, 40]. In fact, in many studies, a direct relationship has now been shown between antibody levels and severity of diseases [41–44]. Similar to these autoimmune diseases, in population screening for celiac disease, antibodies were detected persistently over a 4-year period [45]. Interestingly, nine of the subjects with transient antibodies had villous atrophy, suggesting that this feature develops after chronic immune activation including T-cell response, cytokines and antibody production [39, 45, 46]. Thus, according to these authors, as with type 1 diabetes and thyroiditis, a substantial proportion have transient autoantibodies, but when the autoantibodies persist, the risk of progression to clinical celiac disease is high. As a result, celiac disease-associated autoantibodies are now widely used for disease prediction and diagnosis. Indeed, removal of the antigen, gluten, is currently the therapy of choice for celiac disease [34]. However, due to long-term immunoreactions and severe tissue destruction, gluten-free diets do not result in complete normalization of duodenal lesions in a majority of patients with celiac disease [47]. Therefore, early detection of biomarkers associated with chronic immune activation may result in timely intervention and the prevention of villous atrophy. 

Since earlier studies performed on intestinal T cells [5, 23–26] showed that response to various gliadin and other associated peptides is heterogeneous, we believe that the application of IgG and IgA antibodies against an array of antigens and peptides that includes *α*-,  *γ*-, and *ω*-gliadins, glutenin, WGA, gluteomorphin, prodynorphins, TG, and gliadin-bound TG can not only enhance the detection of celiac disease but may also assist in the early detection of atypical and silent celiac disease. 

Atypical celiac disease, which presents with few or no symptoms, is largely responsible for the increased prevalence of CD today [48]. Celiac disease may be silent or atypical, but it is still a serious disorder [49]. It has been shown that for every recognized case of CD, there are 8 that remain undiagnosed [49], and undiagnosed CD can have very serious consequences. The consequences of undiagnosed CD include not only underachievement [50] and a 5-fold higher risk of non-Hodgkin's lymphoma [51] but also a 4-fold increase in all-cause mortality [52].

Due to some symptomatology overlap between Crohn's disease and CD [12], we applied IgG and IgA measurements against various wheat antigens and associated peptides to the sera of patients with Crohn's disease who were positive for ASCA to examine the occurrence of CD with IBD. In comparison with healthy controls, IgG antibody in the sera of patients with Crohn's disease was found to be highly elevated, foremost against wheat extract (67%), secondly against WGA (50%), prodynorphin third (46%), and then TG (42%), with *P* values being significant against 9 out of 11 tested antigens (Tables 2 and 3). The differences in IgA antibody response against the same array of wheat antigens and peptides used in the study were less significant (Tables 2 and 3), only being significant against 4 of the antigens or peptides: prodynorphin, *α*-gliadin-15, TG, and WGA.

Based on these findings, we propose that for the early detection of immune activation in atypical or silent celiac disease and patients with IBD or Crohn's disease, IgG and IgA antibodies be measured not only against *α*-gliadin and TG, but also against water- and alcohol-soluble components of wheat, WGA, *ω*- and *γ*-gliadin, glutenin, gluteomorphin, and gliadin bound to TG. This may increase the sensitivity and specificity of assays for sufferers not only of classical celiac disease, but also atypical or silent CD as well as patients with IBD who may suffer from gluten sensitivities.

It can be speculated that in addition to gluten-free diets for patients with CD who are ASCA positive, yeast-free diets may also be recommended. If the yeast-free diet along with the gluten-free diet helps patients to get well, then this practice may become an acceptable alternative method of therapy. Further studies are needed in order to compare and measure T-cell and antibody response to these various antigens and peptides simultaneously in patients with normal and abnormal villi.

## Figures and Tables

**Table 1:** Number of specimens with elevated antibodies against 11 tested antigens or peptides at the cutoff point of 0.39 OD.

	IgG						IgA					

	Healthy controls		Gluten sensitive		Crohn's		Healthy controls		Gluten sensitive		Crohn's	
	*n* = 48		*n* = 24		*n* = 24		*n* = 48		*n* = 24		*n* = 24	
	#	%	#	%	#	%	#	%	#	%	#	%
Wheat	9	19	13	54	16	67	5	10	24	100	6	25
*α*-Gliadin 33	1	2	5	21	7	29	2	4	9	38	3	13
*α*-Gliadin 17	0	0	8	33	4	17	9	19	9	38	3	13
*γ*-Gliadin 15	0	0	11	46	9	38	0	0	12	50	8	33
*ω*-Gliadin 17	1	2	10	42	5	21	2	4	11	46	3	13
Glutenin 21	11	23	12	50	6	25	9	19	15	63	3	13
Gluteomorphin	10	21	8	33	8	33	5	10	12	50	4	17
Prodynorphin	0	0	14	58	11	46	4	8	17	71	10	42
Gliadin + TG	1	2	8	33	8	33	0	0	14	58	2	8
TG	0	0	16	67	10	42	0	0	20	83	8	33
WGA	1	2	4	17	12	50	0	0	13	54	3	13

*P* values			0.0004		0.0017				0.0001		0.1262	

TG = transglutaminase.

**Table 2:** IgG antibody expressed as optical density (OD) against wheat and all gliadin, glutenin peptides, exorphins, gliadin-transglutaminase, transglutaminase, and WGA in healthy control subjects and patients with gluten sensitivity and Crohn's disease.

	Wheat (OD)	*α*-Gliadin 33 (OD)	*α*-Gliadin 17 (OD)	*γ*-Gliadin 15 (OD)	*ω*-Gliadin 17 (OD)	Glutenin 21 (OD)	Gluteo-morphin (OD)	Pro-dynorphin (OD)	Gliadin + transglut (OD)	Transglut (OD)	WGA (OD)
Control mean ± SD	0.27 ± 0.12	0.09 ± 0.09	0.08 ± 0.09	0.07 ± 0.08	0.13 ± 0.10	0.29 ± 0.18	0.25 ± 0.18	0.09 ± 0.08	0.06 ± 0.09	0.08 ± 0.10	0.10 ± 0.11

Gluten Sensitive Patient mean ± SD	0.45 ± 0.38	0.18 ± 0.37	0.38 ± 0.53	0.62 ± 0.60	0.37 ± 0.52	0.55 ± 0.52	0.35 ± 0.49	0.64 ± 0.57	0.40 ± 0.50	0.85 ± 0.76	0.36 ± 0.66

*P* values	0.0155	0.1278	0.0053	0.0001	0.0167	0.0104	0.1565	0.0001	0.0013	0.0001	0.0314

Crohn's patient Mean ± SD	0.71 ± 0.57	0.23 ± 0.35	0.22 ± 0.36	0.47 ± 0.53	0.26 ± 0.43	0.29 ± 0.39	0.24 ± 0.34	0.59 ± 0.58	0.34 ± 0.43	0.63 ± 0.65	1.00 ± 1.06

*P* values	0.0005	0.0306	0.0341	0.0006	0.0692	0.4744	0.4621	0.0002	0.0021	0.0002	0.0002

**Table 3:** IgA antibody expressed as optical density (OD) against wheat and all gliadin, glutenin peptides, exorphins, gliadin-transglutaminase, transglutaminase, and WGA in healthy control subjects and patients with celiac and Crohn's disease.

	Wheat (OD)	*α*-Gliadin 33 (OD)	*α* -Gliadin 17 (OD)	*γ*-Gliadin 15 (OD)	*ω*-Gliadin 17 (OD)	Glutenin 21 (OD)	Gluteo-morphin (OD)	Pro-dynorphin (OD)	Gliadin + transglut (OD)	Transglut (OD)	WGA (OD)
Control mean ± SD	0.23 ± 0.15	0.11 ± 0.09	0.25 ± 0.26	0.11 ± 0.06	0.13 ± 0.08	0.23 ± 0.23	0.19 ± 0.16	0.13 ± 0.10	0.06 ± 0.06	0.10 ± 0.07	0.11 ± 0.07

Gluten sensitive Patient mean ± SD	0.89 ± 0.30	0.28 ± 0.37	0.45 ± 0.51	0.71 ± 0.60	0.52 ± 0.54	0.66 ± 0.51	0.45 ± 0.48	0.84 ± 0.56	0.61 ± 0.51	1.01 ± 0.60	1.11 ± 0.95

*P* values	0.0001	0.0195	0.0411	0.0001	0.0009	0.0003	0.0078	0.0001	0.0001	0.0001	0.0001

Crohn's patient Mean ± SD	0.29 ± 0.50	0.13 ± 0.35	0.15 ± 0.33	0.39 ± 0.49	0.11 ± 0.29	0.15 ± 0.28	0.14 ± 0.28	0.44 ± 0.50	0.12 ± 0.25	0.52 ± 0.73	0.40 ± 0.81

*P* values	0.2777	0.3859	0.0935	0.0044	0.3312	0.0992	0.2106	0.0035	0.1544	0.0047	0.0517

## Abbreviations

ASCA: Anti-Saccharomyces cerevisiae mannan antibodies
BSA: Bovine serum albumin
CD: Celiac disease
ELISA: Enzyme-linked immunosorbent assay
F(ab′): Fragment antigen-binding
HSA: Human serum albumin
IBD: Inflammatory bowel disease
IFN-*γ:*: Interferon gamma
Ig: Immunoglobulin
OD: Optical density
TBS: Tris-buffered saline
TG: Transglutaminase
WGA: Wheat germ agglutinin.
